# Danish Cattle Farmers' Experience With Fitness for Transport – A Questionnaire Survey

**DOI:** 10.3389/fvets.2022.797149

**Published:** 2022-03-18

**Authors:** Kirstin Dahl-Pedersen

**Affiliations:** Section for Medicine and Surgery, Department of Veterinary Clinical Sciences, University of Copenhagen, Copenhagen, Denmark

**Keywords:** animal transport, animal welfare, cattle, farmers, fitness for transport, pre-slaughter logistic chain

## Abstract

Worldwide, cattle are transported in great numbers for breeding, fattening and slaughter. Within the European Union, the Council Regulation 1/2005 states that all animals must be fit for transport. Yet, the line between fit and unfit is blurred as the regulation allows for animals that are slightly ill or injured to be transported. However, “slightly ill or injured” lack a clear definition leaving room for individual interpretation of fitness for transport with potential negative implications in terms of both animal welfare and legal certainty. The aim of the present study was to gain an understanding of cattle farmers' experience with and doubt about assessment of fitness for transport-a topic that has received limited scientific attention, despite the important role of farmers in maintaining acceptable animal welfare during transport. The results of the study are based on 119 Danish cattle farmers' answers to a questionnaire survey. The majority of respondents felt they possessed the knowledge and skills required for assessment of fitness for transport. However, a considerable large part of the respondents, one third approximately, reported to be in doubt at least sometimes and likewise one third felt a lack of knowledge at least sometimes. In addition, more than half of the respondents reported that they at least sometimes found it difficult to understand the rules to address the disconnect between on-farm and slaughterhouse decisions and fitness for on-farm slaughter. These results indicate that learning materials, assessment tools and training programs could be helpful for a large group of cattle farmers in order to secure animal welfare during transport. The results also underline the need for further research to clarify what constitutes a “fit” vs. “unfit” animal.

## Introduction

In modern cattle farming, animals are typically transported at least once in their life–to the slaughterhouse. Other reasons for transporting cattle include movements between farms for breeding or fattening. In order to secure acceptable animal welfare during transport it is necessary that the animals are fit for transport (1, 2). Transport can be thought of as a series of stressful events (3), and factors relating both to the individual animal and to the transport conditions might have adverse effects on the welfare of the animal during transport (4–8). Within the European Union (EU), Council Regulation 1/2005 regulates transport of animals (9). The regulation states that all animals must be fit for transport and transported under conditions that will keep them safe from injury and unnecessary suffering. Thus, before being loaded onto a truck fitness for transport of each individual animal must be assessed and farmers and livestock drivers share the legal responsibility for this assessment. However, fitness for transport can be quite difficult to assess. The term “fit for transport” is somewhat vaguely defined in the legislation. Indeed, a list of clinical conditions that should without doubt cause an animal to be judged as unfit is provided e.g., animals in the last tenth of their pregnancy, new-born animals with unhealed navels and animals with prolapses. Yet, the line between fit and unfit is blurred as the regulation allows for animals that are slightly ill or injured to be transported if the transport will not cause additional suffering. What “slightly ill or injured” or “additional suffering” means is not further defined in the regulation. This lack of definition leaves room for individual interpretation of fitness for transport with potential negative implications in terms of both animal welfare and legal certainty. A report from the European Commission (10) on the impact of Council Regulation 1/2005 described “recurring examples of poor compliance such as transport of unfit animals”. Also outside the EU, assessment of fitness for transport is a topic of concern. One of the recommendations that came out of an expert consultation on management of cull dairy cows involving farmers, veterinarians and experts in animal transport in Canada was to improve the ability of personnel to assess animal condition before loading (11).

Cattle farmers have to make decisions about fitness for transport on a regular basis. In Denmark, approximately 470,000 cattle are transported to slaughter at a national slaughterhouse each year, the majority is young beef bulls and cull dairy cows (12). In addition, approximately 80,000 cattle are exported, the majority is unweaned calves destined for fattening in other EU countries and pregnant heifers, some of which are bound for distant destinations e.g., Russia (12). As such, farmers play an important role in securing animal welfare in the pre-slaughter logistic chain, yet relatively little is known about their knowledge, doubts and decision-making process in regards to animal transport. During the last decades, farmers' views, perceptions and knowledge of different aspects of farm animal welfare have received increasing scientific attention as reviewed by Balzani and Hanlon (13). However, few studies have focused on management related to animal transport and assessment of fitness for transport (14–17).

Danish farmers have since the enforcement of Council Regulation 1/2005 been introduced to animal transport, management related to transport and assessment of fitness for transport as part of their education (18), but no continuing education programs or training directed at farmers who were educated before 2005 currently exist. A study by Dahl-Pedersen et al. (14) showed that farmers, livestock drivers and veterinarians at best agreed moderately when assessing dairy cow fitness for transport in relation to locomotion score, and farmers agreed least both within their own group and with the two other groups, the livestock drivers and the veterinarians.

The aim of the present study was to gain an understanding of cattle farmers' experience with and potential doubt about assessment of fitness for transport. Insight into the challenges farmers might face in their daily work is a prerequisite for pointing future research in the right direction and for developing relevant learning materials and training programs.

## Materials and Methods

### Recruitment of Participants and Data Collection

The study population was cattle farmers in Denmark. Convenience sampling was used to recruit respondents. An invitation was distributed in the two Danish Facebook groups “Danish Producers of Beef Calves” and “Danish Beef Cattle Breeding” with a total of 2,697 member, primarily cattle farmers. An invitation was also distributed through the Danish Facebook group “Veterinarians interested in Cattle Diseases” (Danish veterinarians) encouraging the 795 members to pass on the questionnaire to clients. Through the Central Farm Animal Register (Det Centrale Husdyrregister, Danish Veterinary and Food Administration, www.chr.dk), an official national database of all farm animal herds in Denmark 545 email-addresses belonging to cattle farmers were retrieved. An invitation to participate was sent to them all. The list of emails-addresses was handled in accordance with GDPR rules regarding secure storage and destruction.

### The Questionnaire

The questionnaire consisted of 18 questions; the first four were demographic, questions five to seventeen focused on shipping routines and experience with cattle fitness for transport. As a last question, respondents were asked if they had anything they wished to add (see Table 1).

**Table 1:** List of questions and answer categories in the questionnaire sent to Danish cattle farmers in order to examine their experience with assessment of fitness for transport.

	**Questions**		**Answer categories**
1	Are you the owner of the farm or an employee?	A	Owner
		B	Employee
2	How old are you?		
3	What is your educational background?	A	Trained farmer
		B	Trained animal keeper
		C	Other education
		D	No education
4	For how many years have you been working with cattle?	A	Less than a year
		B	1–3 years
		C	3–10 years
		D	More than 10 years
5	How often do you ship animals?	A	Several times a week
		B	Once a week
		C	1–4 times a month
		D	Once a month or less
6	What type of animals do you most frequently ship?	A	Calves younger than 3 months
		B	Calves and young animals between 3 and 24 months
		C	Adult cattle
7	What is most often the purpose of shipping?	A	Fattening or breeding, national
		B	Slaughter, national
		C	Fattening or breeding, export
		D	Slaughter, export
8	How familiar are you with the current legislation regarding cattle fitness for transport?	A	I am not familiar with the legislation
		B	I am familiar with legislation, but do not remember any specific rules
		C	I am quite familiar with the legislation and remember several specific rules
		D	I am very familiar with the legislation and remember most rules
9	Do you find it difficult to understand the rules regarding cattle fitness for transport?	A	Very often
		B	Often
		C	Some times
		D	Rarely
		E	Never
10	How did you acquire you knowledge about cattle fitness for transport?	A	Through my education
		B	I was taught by a colleague
		C	I was taught by a livestock driver
		D	I was taught by a veterinarian
		E	It is self-taught
11	Do you think you lack knowledge regarding assessment of cattle fitness for transport	A	Very often
		B	Often
		C	Some times
		D	Rarely
		E	Never
12	On what do you base your assessment of fitness for transport?	A	An examination of the animal
		B	Observation of the animal from a distance
		C	Earlier observations
		D	Earlier observations and an examination
		E	I do not make an assessment
13	How often are you in doubt about an animal's fitness for transport?	A	Very often
		B	Often
		C	Some times
		D	Rarely
		E	Never
14	Have you ever shipped an unfit animal?	A	Very often
		B	Often
		C	Some times
		D	Rarely
		E	Never
15	What do you do with an animal if you doubt whether it is fit for transport?	A	Try to ship it anyway
		B	Ask for the driver's opinion
		C	Call the vet for an opinion
		D	Place the animal the stable and wait for the next shipping opportunity
		E	Place the animal in a sick pen and wait for the next shipping opportunity
		F	Euthanize the animal
16	Which type of clinical condition are you most doubtful about?	A	Lameness
		B	Body condition score
		C	Wounds
			
		D	Udder lesions
		E	Other conditions
17	Do you feel able to assess whether the clinical condition a slightly ill or injured animal may worsen during transport?	A	Very often
		B	Often
		C	Some times
		D	Rarely
		E	Never
18	Do you have anything to add?		

It was voluntarily and anonymous to participate and it was possible to quit at any given point. Time required to answer to questionnaire was <10 min. Only questionnaires for which questions 1–17 had been completed were include in the study, while question 18 was optional and non-completion did not lead to exclusion from further analysis. The study was carried out in May and June 2020.

### Statistics

Descriptive statistical analyses were conducted and the results are presented as percentages, and when appropriate mean and range. As for the open question (question 18), the percentage of respondents answering was calculated and a thematic evaluation was performed to capture the most common categories of comments.

### Limitations

When interpreting the results of this study it should be kept in mind that the sample size of the study was rather small–Denmark has around 2,500 dairy farms plus a number of smaller beef producers–and the sample was also not randomly selected. It is possible that participants with a special interest in the topic are overrepresented. In addition, web-based questionnaire surveys have a number of known limitations such as self-reporting-bias, but is nevertheless an accepted way of investigating tendencies (19, 20).

## Results

A total of 119 completed questionnaires were included. It was not possible to calculate a response rate, since the questionnaire was partly distributed via Facebook.

### Demographics

The majority of respondents were farm owners (85%). Mean age was 44 years (range 18–70), and the majority (88%) had more than 10 years of experience with cattle, only 3% had <3 years of experience. Most were formally educated farmers (83%).

### Transport Routines

The majority of respondents (68%) shipped animals between 1 and 4 times per month, 21% shipped less frequently, and 11% shipped more frequently. Adult cattle was the most frequently transported group of animals (58%), and the most common purpose for shipping was slaughter at a national slaughterhouse (70%).

### Experience With Fitness for Transport

Most of the respondents (85%) indicated that they were familiar with the legislation regarding cattle fitness for transport and knew several or most rules. However, more than half (59%) indicated that they very often, often or sometimes found it difficult to understand the rules (see Table 2).

**Table 2:** Questions 9, 11, 13, 14 and 17 from the questionnaire sent to Danish cattle farmers in order to examine their experience with assessment of fitness for transport.

	**Very often**	**Often**	**Sometimes**	**Rarely**	**Never**
Do you find it difficult to understand the rules regarding cattle fitness for transport?	9%	30%	20%	37%	4%
Do you think you lack knowledge regarding assessment of cattle fitness for transport?	3%	11%	20%	52%	14%
How often are you in doubt about an animal's fitness for transport?	1%	2%	29%	60%	8%
Have you ever shipped an unfit animal?	0%	2%	13%	53%	33%
Do you feel able to assess whether the clinical condition of a slightly ill or injured animal may worsen during transport?	27%	41%	20%	12%	0%

*N = 119*.

The respondents were asked how they learnt about cattle fitness for transport and were allowed to give more than one answer: 60% responded taught by a veterinarian, 60% responded taught by a livestock driver, and 60% responded to be self-taught, 32% responded through formal farmer education, and 10% responded taught by a colleague. Two thirds of the respondents rarely or never felt they lacked knowledge regarding assessment of fitness for transport, while one third felt they at least sometimes lacked knowledge (see Table 2).

Almost half of the respondents (49%) based their assessment of a specific animal on an examination just before loading, while 35% would combine such examination with the history of the animal, 16% would not include an examination, but base their assessment on either observation of the animal from a distance or the history of the animal. None responded that they made no assessment at all. Fourteen percent reported that they often or sometimes had shipped an unfit animal, and 86% reported that they never or rarely had shipped an unfit animal. None reported to have shipped an unfit animal often (see Table 2).

### Doubt About Fitness for Transport

Only three percent of the respondents reported to experience doubt about an animal's fitness for transport very often or often. A little less than a third of the respondents (29%) reported to experience doubt sometimes, while the majority (68%) reported that they never or rarely experienced doubt (see Table 2).

Lameness was by far the condition that respondents reported would most often lead to doubt about fitness for transport. In total, 87% reported this to be the most difficult condition to assess (see Table 3). Two thirds of the respondents reported that they very often or often felt able to assess whether or not an animal's condition would worsen during transport, but one third sometimes or rarely felt able to assess this (see Table 2).

**Table 3:** Question 16 in the questionnaire sent to Danish cattle farmers in order to examine their experience with assessment of fitness for transport.

	**Lameness**	**Wounds**	**Low body condition score**	**Udder lesions**	**Other conditions**
Which type of clinical condition do you find makes it most difficult to assess fitness for transport?	87%	2%	4%	0%	7%

If in doubt about the fitness for transport of an animal the majority of respondents would wait for the next shipping opportunity and meanwhile place the animal either in a sick pen (14%) or in its usual barn section (32%). One out of four would ask for the livestock driver's opinion and one out of five would ask the for veterinarian's opinion. Only one percent would try to ship it anyway.

As a final question, the respondents were asked if they had anything to add. Forty-nine respondents (41%) provided additional statements. Thirty-three of the comments related to just two issues: 1) 41% of the respondents that added comments found assessment of fitness for transport too subjective resulting in many different opinions from different professionals. Thus, the respondents expressed fear of being reported to the police by the veterinary authorities at the slaughterhouse despite having sought advice from their own veterinarian before loading. The respondents stated that they in particular valued the opinion of the livestock driver; 2) 27% of the respondents that added comments found it unreasonable that animals unfit for transport due to e.g., some degree of chronic lameness but otherwise healthy could not either be transported to the nearest slaughterhouse with special provisions or be slaughtered at the farm and then shipped to the slaughterhouse like acutely injured animals.

## Discussion

The present study focuses on cattle farmer's experience with and doubt about assessment of fitness for transport. This topic has received limited scientific attention, despite the important role of farmers in maintaining acceptable animal welfare during transport. The majority of respondents felt they possessed the knowledge and skills required for assessment of fitness for transport. However, approximately, one third of the respondents reported to be in doubt at least sometimes and, likewise, one third felt a lack of knowledge at least sometimes. More than half of the respondents reported that they at least sometimes found it difficult to understand the rules. These results indicate that learning materials, assessment tools and training programs could be helpful for a large group of cattle farmers.

The respondents reported that they would ask the livestock driver for advice regarding fitness for transport of a specific animal more often than they would ask the veterinarian. There can be several reasons for this. For instance, it is convenient as the driver is already at the farm and advice is free of charge and perhaps more importantly, the livestock driver has unique experience since he–unlike farmer and veterinarian-sees the animals at both loading and unloading. Like farmers, livestock drivers play an important role in securing animal welfare during transport, yet very few studies have focused on this group of professionals (21–24). Herskin et al. (21) did a study on Danish livestock drivers and found that 35% of the respondents at least frequently experienced doubt about the fitness for transport of a cow and that only 52% could answer two specific questions about cattle fitness for transport correctly. Livestock drivers often relay on peer-to-peer training rather than formal education when learning about animal welfare during transport (21, 23). Dahl-Pedersen et al. (14) showed that there was only moderate agreement among and between farmers, livestock drivers and veterinarians regarding fitness for transport of dairy cows in relation to locomotion scoring. Taken together, results from the present study as well as the abovementioned studies suggest that future training programs should include veterinarians, farmers and livestock drivers in order to seek to standardize the assessment of fitness for transport and hopefully agree on more consistent views on “fit” vs. “unfit”.

In the present study, the vast majority of farmers selected lameness as the type of clinical condition that most often resulted in doubt in relation to assessment of fitness for transport. Similar results were found in a study of Danish sow farmers (16). Lameness is one of the most prevalent health problems in cattle production, in particular in the dairy industry. Dahl-Pedersen et al. (25) reported that 31% of cull dairy cows were lame before transport to slaughter. Several studies have shown that farmers in general underestimate the prevalence of lameness in their herds or have difficulties recognizing lameness (26–28). It is therefore not surprising that lameness is by far the one condition farmers find most difficult to assess. These results clearly demonstrate that lameness should be a focus point in future learning materials and training programs for farmers. However, it is important to remember that lameness is just one component of assessment of fitness for transport and other clinical conditions can be equally important. In order to gain a better understanding of the complex task of assessing fitness, future research should include other conditions as well, e.g., body condition score.

One major common frustration among the respondents was what they considered a lack uniformity and predictability in assessment of fitness for transport done by veterinarians, in particular the veterinarians doing live inspections at slaughterhouses. The farmers found it very hard to accept that for instance a cow whose history they knew well and whose fitness for transport they had discussed with the livestock driver and/or the veterinarian before loading could risk ending up being judged unfit for transport upon arrival to the slaughterhouse. Some farmers mentioned that due to fear of misjudging they were not willing to take the slightest chance, which in some cases refrained them from shipping specific animals and instead lead them to kill the animals at the farm. Even short distance transport can have adverse effects on the clinical condition of cattle, e.g., dairy cows may become lame or more lame during transport (6). Yet in a Canadian study, dairy farmers were strongly confident that the clinical condition of their cows would not change during transport (17). In addition, it has been shown in other studies that farmers and veterinarians have different attitudes toward e.g., animal pain (29, 30). This could explain some of the frustration felt by farmers when the veterinarian at the slaughterhouse overruled their own assessment of fitness. Several respondents suggested that an official homepage from the veterinary authorities with abundant examples of different conditions making an animal fit or unfit for transport would be beneficial-preferably with pictures and film recordings the farmer could use for reference and discuss with the livestock driver and veterinarian. However, an official homepage would not necessarily solve the problem with slaughterhouse veterinarians overruling the assessment done by the farmer if the clinical condition of the animal has deteriorated during transport. Also, the authorities could potentially have reservations regarding launching material that could be interpreted as a bulletproof fitness checklist. Future research is needed to compare assessments done by farmers and slaughterhouse veterinarians in more detail in order to identify common inconsistencies.

According to Danish rules, acutely, seriously injured animals must not be transported, but can–if treatment is not an option, and they are otherwise healthy–be slaughtered at the farm and the carcass can then be transported to the nearest slaughterhouse for further processing. However, animals not acutely injured, but suffering from e.g., chronic lameness making them unfit for transport cannot be slaughtered at the farm, but must either be treated or killed and discarded (31). The respondents found these rules highly unreasonable and argued that the animal would have better welfare if transported for a short time to a slaughterhouse instead of being left to recover at the farm, potentially for a long time and potentially without chance of getting better. In addition, the respondents emphasized that they felt wrong about meat going waste if the animal had to be killed and discarded, and importantly this would cause the mortality rate for the herd to go up. Herd mortality rate is a key figure that the Danish veterinary authorities monitor. If it raises above a certain level farmers will automatically receive more mandatory veterinary inspections (32). Livestock drivers expressed similar view regarding welfare and waste of meat (21). These are very concrete and practical considerations, which future training programs and learning materials must incorporate. It can be discussed whether this is a matter of timely culling strategies, rather than a matter of assessing fitness for transport. However, to the farmers these issues are probably highly interconnected. Future studies combining farmers' views on culling and fitness for transport could provide better understanding of this complex decision making process. In addition, the whole issue of whether or not chronically diseased animals should be considered for human consumption warrants further discussion.

## Conclusion

The present study focuses on cattle farmer's experience with and doubt about fitness for transport. This topic has previously received limited scientific attention, despite the important role of farmers in maintaining acceptable animal welfare during transport.

The aim was to gain an understanding of the potential challenges cattle farmers encounter when assessing cattle fitness for transport. Such understanding is a prerequisite for developing relevant learning materials and training programmes in the future. The results point out important questions to address in future research e.g., how farmers can increase their ability to assess lameness in relation to fitness for transport and how farmers, livestock drivers and veterinarians can align their understandings of fitness for transport in order to secure animal welfare and legal certainty. The results also underline the need for further research to clarify what constitutes a “fit” vs. “unfit” animal.

## Data Availability Statement

The raw data supporting the conclusions of this article will be made available by the authors, without undue reservation.

## Ethics Statement

The study was approved by the Research Ethics Board of Section for Medicine and Surgery, Department of Veterinary Clinical Sciences, University of Copenhagen, Copenhagen, Denmark.

## Author Contributions

The author confirms being the sole contributor of this work and has approved it for publication.

## Conflict of Interest

The author declares that the research was conducted in the absence of any commercial or financial relationships that could be construed as a potential conflict of interest.

## Publisher's Note

All claims expressed in this article are solely those of the authors and do not necessarily represent those of their affiliated organizations, or those of the publisher, the editors and the reviewers. Any product that may be evaluated in this article, or claim that may be made by its manufacturer, is not guaranteed or endorsed by the publisher.
