# Erratum: Danish Cattle Farmers' Experience With Fitness for Transport — A Questionnaire Survey

**DOI:** 10.3389/fvets.2022.902717

**Published:** 2022-04-06

**Authors:** Frontiers Production Office

**Affiliations:** Frontiers Media SA, Lausanne, Switzerland

**Keywords:** animal transport, animal welfare, cattle, farmers, fitness for transport, pre-slaughter logistic chain

Due to a production error, the **Ethics Statement** section of the article was erroneously removed. The section appears below.

“**Ethics Statement**

The study was approved by the Research Ethics Board of Section for Medicine and Surgery, Department of Veterinary Clinical Sciences, University of Copenhagen, Copenhagen, Denmark.”

The publisher apologizes for this mistake. The original article has been updated.

## Publisher's Note

All claims expressed in this article are solely those of the authors and do not necessarily represent those of their affiliated organizations, or those of the publisher, the editors and the reviewers. Any product that may be evaluated in this article, or claim that may be made by its manufacturer, is not guaranteed or endorsed by the publisher.

